# Living with a giant inguinoscrotal hernia for 35 years—a case report

**DOI:** 10.1093/jscr/rjab458

**Published:** 2021-10-28

**Authors:** Pradeep Khatiwada, Amrit Devkota, Sagar Panthi, Srista Manandhar, Dipendra Sharma, Sunit Chhetri, Chet Bahadur Ranabhat, Suresh Shah, Bhawani Khanal

**Affiliations:** Department of General Surgery, B.P. Koirala Institute of Health Sciences, Dharan 56700, Nepal; Department of General Surgery, B.P. Koirala Institute of Health Sciences, Dharan 56700, Nepal; Department of General Surgery, B.P. Koirala Institute of Health Sciences, Dharan 56700, Nepal; Department of General Surgery, B.P. Koirala Institute of Health Sciences, Dharan 56700, Nepal; Department of General Surgery, B.P. Koirala Institute of Health Sciences, Dharan 56700, Nepal; Department of General Surgery, B.P. Koirala Institute of Health Sciences, Dharan 56700, Nepal; Department of General Surgery, B.P. Koirala Institute of Health Sciences, Dharan 56700, Nepal; Department of General Surgery, B.P. Koirala Institute of Health Sciences, Dharan 56700, Nepal; Department of General Surgery, B.P. Koirala Institute of Health Sciences, Dharan 56700, Nepal

## Abstract

In this modern era, giant inguinoscrotal hernias are very rare to experience in a medical career. We discuss a case of a 65-year-old man with a history of an inguinoscrotal hernia with progressive growth for the past 35 years. On examination, he had a 20 cm × 15 cm non-reducible swelling with multiple ulcers over the skin surface extending to the mid-thigh with otherwise no other bladder and bowel complications. These large hernias pose a different set of surgical problems. Open surgery was performed, hernial sac opened, contents reverted and left orchidectomy were done with scrotal reconstruction. The defect was closed with Vicryl 1-0 over the muscle layer and the skin was stapled. Daily wound care was provided. Besides, this case also compels us to explore possible reasons for the occurrence of such potentially dangerous surgical problems in low-to-middle income countries (LMIC).

## INTRODUCTION

Giant inguinal hernias are hernias extending below the midpoint of the inner thigh in a standing position [1]. Previously, it was defined as a hernia bigger than the size of the head of the same individual [2]. Besides causing a different kind of surgical problems during management, they also reflect the extent of neglect and awareness about potentially dangerous surgical problems in low-to-middle income countries (LMIC), like Nepal. HICs are unlikely to encounter such gigantic hernias, but in LIMCs it is important to be prepared to deal with such hernias when encountered. We discuss a rare case of a giant inguinoscrotal hernia with history of progressive growth for 35 years and its management in our tertiary hospital.

## CASE REPORT

A 65-year-old male presented to the emergency with complaints of a massive swelling over the inguinal region and multiple wounds over its skin surface. The patient initially noticed a cricket ball-sized swelling 35 years back and paid no attention, as it was painless and reducible. Then, the swelling increased itself to the present size in 15 days. It became non-reducible but remained painless. Multiple non-discharging ulcers appeared over the scrotal skin. Patient deferred any medical attention due to his financial condition and lack of confidence toward surgical system. Then only, the patient sought help in a local health post and was referred to our hospital. He had normal bladder and bowel habit and no history of chronic cough, constipation, prostatism or any significant co-morbidity.

**Figure 1:**
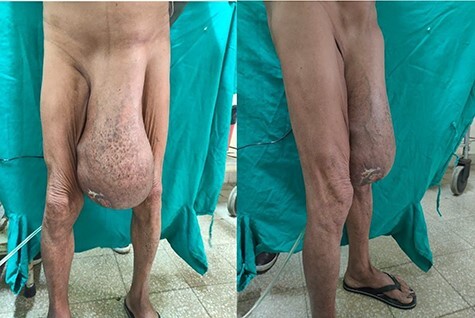
At the time of presentation.

On examination, an inguinoscrotal swelling of 20 × 15 cm^2^ extending below the midpoint of the thigh was observed. The penis was visible but both testicles were impalpable. Dilated and tortuous veins along with multiple non-discharging ulcers were visible over the skin surface (Fig. 1). Cough impulse was present, whereas a tympanic note was heard on percussion.

**Figure 2:**
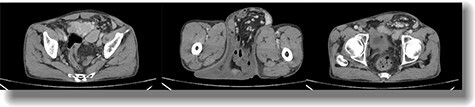
Axial postcontrast CT of the abdomen at the level of pelvis showing a defect in the peritoneal lining in the left inguinal region with protrusion of bowel loops, accompanying mesentery and vessels into the left scrotal sac via inguinal canal suggestive of left inguinoscrotal hernia.

Preoperatively, ultrasonography confirmed it as a left-sided inguinoscrotal hernia with right minimal hydrocele. Computed tomography (CT) scan revealed the contents to be small intestine along with omentum, cecum, appendix, part of the transverse colon, sigmoid colon and their mesocolon (Fig. 2). Echocardiography and pulmonary function test were done revealing mild TR with Grade I Left Ventricular Diastolic Dysfunction and mild (GOLD classification) obstructive lung disease respectively. Required blood tests were done deeming the patient fit for surgery.

**Figure 3:**
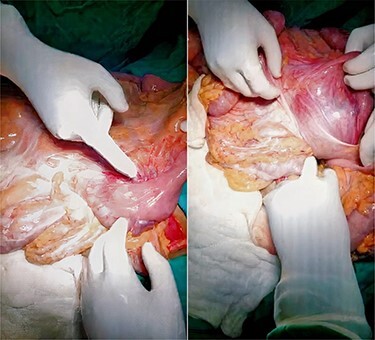
Intraoperative picture showing hernia contents.

During operation, skin crease incision was made two finger-width above pubic tubercle and later extended to the scrotum and deepened to the subcutaneous tissue. The hernial sac was opened and abdominal contents were reduced to the abdomen without any complication (Fig. 3). Atrophic left testis was excised and scrotoplasty was done. Biomesh of 15 × 15 cm^2^ was used to close the defect. After fixing the mesh with prolene 3-0 RB to pubic tubercle, inguinal ligament, conjoint tendon and transverse abdominis muscle, external oblique aponeurosis was closed with vicryl 1-0 RB. The defect was closed without any undue tension, and no resection of the bowel or reconstructive procedure of the abdominal wall was attempted. A drain was kept in the scrotum and below external oblique layer to prevent seroma formation. Skin was closed with a stapler.

**Figure 4:**
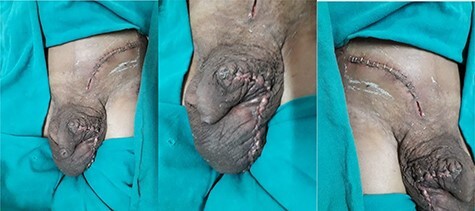
Picture showing incision wound on fourth postoperative day.

Mild discharge from the incision site was observed on the third postoperative day with mild tenderness on the surgical site and fever documented 101 F for 1 day without local rise in temperature. Scrotal support was given and daily coconut dressing was done. The patient was treated with intravenous antibiotics and discharged on oral antibiotics. At discharge, the wound was healthy and vitals were stable (Fig. 4). He did not mention any difficulties, and no complications were noted during his follow-up on the 14th day and 30th day.

## DISCUSSION

Management of giant inguinal hernias presents several specific problems. The size itself can interfere with locomotion and other daily activities. Penis can be buried inside the scrotum causing urine to dribble over the scrotal skin, causing excoriation, ulceration and secondary infection and also affect sexual life [3]. Skin congestion due to venous and lymphatic edema further aggravates the process. Elongation of the ipsilateral spermatic cord possesses the risk of testicular torsion. The testis is often atrophic and unsalvageable. The possibility of complications like intestinal obstruction, incarceration and strangulation is increased and can have fatal outcomes [4].

The common contents of an inguinal hernia are omentum and small bowel; although, stomach, cecum, appendix, sigmoid colon, urinary bladder, ovaries and even the entire mesenteric small bowel and colon have been reported [5–7].

In general, repairing giant inguinal hernias with significant loss of domain can arise serious physiological complications. The increase in intra-abdominal pressure after reduction pushes up the diaphragm and can cause respiratory failure and pneumonia in addition to wound dehiscence or recurrence [8, 9]. A third problem of large residual scrotal skin was encountered during the operation.

As decreasing the bulk of contents is seen to improve prognosis, two methods are proposed. Preoperatively, the use of elemental diets to reduce fecal residues and GI secretion is suggested [10]. Also, a more effective and common practice is resection of the omentum, small bowel or colon, though, it might subject the patient to further intraoperative and postoperative complications [4, 7, 10, 11] For increasing the room of abdominal cavity, older techniques like phrenicectomy [12], iatrogenic incisional hernia [13] and musculoskeletal flaps [11] are no longer used.

Merret *et al*. [14] have reported a case of giant inguinal hernia which was successfully managed by creating a midline anterior wall defect, covering both the hernial and the midline defect with marlex mesh and then strengthening the midline mesh by a rotation flap of the inguinoscrotal skin, which would have been otherwise discarded.

Recurrence is common in conventional repairs [15]. Many surgeons in the past have advocated mesh-free repair [7], but most recent surgeons prefer the use of mesh.

Some authors discourage the scrotal reconstruction as a safety precaution so that the contents can be temporarily reverted back into it if severe respiratory failure develops in early postoperative days [4]. Still, for cosmetic reasons, scrotum reconstruction can be performed in single or double stages [1, 13].

Herniorrhapy is one of the most frequently performed general surgical operations worldwide. However, most LMICs are less capable to deliver early intervention for common general surgery issues such as an inguinal hernia to the public, resulting in considerable morbidity and mortality. A cluster randomized, cross-sectional household survey performed in a LMIC, 31% of the respondents were not able to have surgery due to unavailability of surgical services, fear or mistrust toward surgical system (31%) and inability to afford care (21%). 20% were unable to work as previous or perform self-care due to their hernia [16]. Similar study in Guatemala demonstrated that most patients with an incarcerated inguinal hernia (56%) did not seek medical attention because of family obligations. Most (56%) did not have any formal education. They concluded that emergent hernias are likely the result of patient-related issues rather than health care system limitations [17].

## CONCLUSION

The management of giant scrotal hernia has its unique challenges that require proper preoperative vigilance, intraoperative acumen and postoperative care. Early detection and treatment can prove significant in improving the quality of life of rural patients.
